# A Word on Dysentery

**Published:** 1854-01

**Authors:** W. S. Linn

**Affiliations:** Chili, Ill.


### IOWA MEDICAL JOURNAL.

VOL. I. KEOKUK, IOWA, JANUARY, 1854. NO. 6.

### ORIGINAL COMMUNICATIONS.

### *A word on Dysentery.*

BY W. S. LINN, M. D., CHILI, ILL.

As much has, of late, been published in Medical Journals regarding this disease, I have ventured to add a word, which I hope will not be deemed out of place. Dysentery has prevailed here to a considerable extent during the past season, in its most perplexing character. Not that it proved fatal in any case, but the symptoms at first developed, were so slight that it was difficult for the medical attendant to decide whether to give any thing at all or leave the case to nature. Its attacks were insidious, and I soon learned to regard them with a secret fear. All the symptoms seemed to be measurably masked and very mild; consequently demanding lenient restorative means. But with any treatment primarily adopted, the approach towards a healthy standard was exceedingly tardy. In a few instances, the questions of the friends as to the probability of the patient's recovery, were answered favorably without hesitation, so secure did I feel in predicting a speedy return of health; and I am sure I was warranted in so doing from the character of the symptoms and every before-observed precedent. I was, however, disappointed, time and again, although the favorite means of our standard authors, were fully tried in the first ten or twelve cases. Almost every thing that has been suggested as being of salutary effect was brought into requisition, and failed. The characteristics of the cases were such as are prominent in bloody flux, viz: tormina, tenesmus, frequent diminutive and bloody stools, with slime. There was no continuous fever, very little thirst, tongue invariably red and covered with bilious coating of immoderate density. In only one or two cases was there any vomiting or much prœcordial distress. There was diffuse tenderness over most of the abdomen, particularly in the hypogastric and right hypochondriac regions. The loss of appetite was complete; indeed there was an unusual loathing of food, till the fifth or sixth day, when it returned with an intensity which the state of the patient rendered it imprudent to gratify. The skin was, in every instance cold; but the system evinced no signs of periodical congestion. The pulse was weak, thready and irregular. The patients usually enjoyed immunity from a desire to go to stool during the night, at which time, those who are laboring under this disease, generally experience the most of this trouble. The urine was scanty and high-colored, highly offensive and irritating to the neck of the bladder and urethra.

The treatment was, as before stated, varied and for a time inefficient. The mercurial plan was tried, assisted by an occasional dose of caster oil. I had recourse also to large doses of opiates, with the warm foot-bath and the usual auxiliaries. After trying, in vain, such plans of treatment, I resorted to the use of injections of slippery elm mucilage, to which I added a small quantity of morphia. These were administered cold, and as often as once in four hours. By this means, I soon had the satisfaction to see the pain relieved and the small wasting discharges stopped. In the place of the mercurial, I substituted the nitric acid. I also made use of warm salt water baths to the body, hot mustard plasters to the feet, with a weak alcoholic solution of camphor to the abdomen. In some of the mildest cases, instead of the nitric acid, I gave finely grated horse-radish, and was pleased with its effects.

REMAKKS.—During the whole prevalence of the disease, there was a most remarkable wasting of the system, considering the mildness of the symptoms. The attacks usually occurred after some imprudence, such as eating to excess, of fruits or other substances difficult of digestion. The inflammation was subacute, and generally appeared in those whose systems showed a scrofulous tendency.

